# Barriers and facilitators to the national scale‐up of a preterm standardised parenteral nutrition system: A mixed‐methods evaluation

**DOI:** 10.1002/jpr3.70213

**Published:** 2026-07-31

**Authors:** Sarah Fenton, Ann‐Marie Brennan, Aoife Fleming, Vicki Livingstone, Brendan Murphy, Aileen Murphy, Eugene Dempsey, Ciara Heavin

**Affiliations:** ^1^ The Irish Centre for Maternal and Child Health Research (INFANT Research Centre) University College Cork Cork Ireland; ^2^ Pharmacy Department Cork University Hospital Cork Ireland; ^3^ Department of Clinical Nutrition & Dietetics Cork University Maternity Hospital Cork Ireland; ^4^ School of Pharmacy University College Cork Cork Ireland; ^5^ Department of Paediatrics & Child Health University College Cork Cork Ireland; ^6^ Department of Paediatrics University Hospital Waterford Waterford Ireland; ^7^ Department of Economics, Cork University Business School University College Cork Cork Ireland; ^8^ Department of Neonatology Cork University Maternity Hospital Cork Ireland; ^9^ Cork University Business School University College Cork Cork Ireland

**Keywords:** health service research, implementation science, neonatal

## Abstract

**Objective:**

Standardising parenteral nutrition (PN) care for preterm infants is recommended to enhance safety, outcomes and efficiency, however implementation at scale remains challenging. In 2021, Ireland scaled‐up a standardised parenteral nutrition (SPN) system comprising SPN formulations and dosing protocol. We evaluate national scale‐up to identify contextually relevant factors to optimise implementation.

**Methods:**

A sequential explanatory mixed‐methods process evaluation was conducted. Phase 1 comprised a cross‐sectional survey of healthcare professionals (HCPs) across nine neonatal units (NUs), alongside analysis of PN purchasing data. Phase 2 included a focus group with the national implementation team and semi‐structured interviews with HCPs from three NUs. Qualitative data were analysed using framework analysis guided by the Consolidated Framework for Implementation Research.

**Results:**

For Phase 1, we analysed 158 survey responses (27.6% response rate). All NUs had adopted the SPN system; 95% HCPs reported ‘Always’ or 'Often’ using the protocol. SPN accounted for 95% of PN purchased nationally, increasing from 56% pre‐implementation. Usability and sustainment scores were significantly higher among trained HCPs (*p* = 0.026; *p* < 0.001). Protocol adaptations were reported by 21%, and 16% reported receiving no training. Phase 2 (1 focus group; 16 interviews) developed seven themes describing factors influencing scale‐up. Key enablers included the perceived relative advantage of the intervention and strong national networks. Barriers included local policy misalignment, limited evidence in specific populations and hierarchical decision‐making.

**Conclusion:**

Scale‐up of SPN interventions is achievable in practice, however contextual factors influenced how the intervention was used. System‐level infrastructure including training and ongoing monitoring is required to optimise implementation and equitable sustainment.

## Abstract (highlights)

**What Is Known:**

- Standardising preterm parenteral nutrition (PN) care, including using standardised PN (SPN) formulations, guidelines and protocols is recommended to improve safety, efficiency and clinical outcomes.
- Implementation of these interventions remains suboptimal, scale‐up of SPN formulations has been associated with poorer outcomes, and there is limited evidence on how to effectively implement and scale neonatal nutrition interventions.

**What Is New:**

- Scale‐up of an SPN intervention across multiple neonatal units is achievable in practice with high reported adoption and sustainment.
- An implementation science approach enabled identification of behavioural and contextual factors influencing how the intervention was used in practice, providing insights that would not have been revealed through traditional evaluation approaches.
- Findings highlight that successful scale‐up requires system‐level infrastructure including training and audit to optimise fidelity and equitable sustainment.
- Future preterm PN research should incorporate prospective evaluation of implementation alongside effectiveness to better understand how complex PN interventions are adopted, adapted and sustained in the neonatal setting.

## 1 INTRODUCTION

Early nutrition in preterm infants is critical for short and long‐term health outcomes, yet optimising delivery is challenging.^1^ Consensus guidelines recommend standardising nutritional care, through standardised parenteral nutrition (SPN) formulations, guidelines and protocols to enhance safety, efficiency and clinical outcomes.^2^, ^3^, ^4^, ^5^ However, adoption is suboptimal,^6^, ^7^, ^8^ in part constrained by access to suitable SPN formulations and tools that translate nutritional recommendations into practice.^6^

Scale‐up, the integration of effective interventions into routine practice and policy, offers an opportunity to enhance uptake of interventions.^9^ For SPN, this may reduce healthcare costs and improve continuity of care during hospital transfer.^10^ However, SPN scale‐up can produce unintended consequences, with a recent report of increased morbidity and mortality associated with higher‐than‐intended macronutrient delivery.^11^ These findings highlight that parenteral nutrition (PN) is a complex intervention^12^, ^13^ and evidence to guide real‐world implementation and scale‐up is required.^9^ However, this evidence remains limited for the dynamic neonatal intensive care setting.^14^

In 2021, Ireland scaled‐up a multimodal preterm SPN system from one neonatal unit (NU) to all maternity‐based units that use PN nationally.^15^ This system comprises SPN formulations and a dosing protocol that standardises SPN prescribing and breastmilk fortification (BMF) based on an infant's enteral feed volume (EFV).^16^, ^17^ This study presents an evaluation of this national scale‐up, examining barriers and facilitators to generate contextually relevant insights to optimise implementation and inform future scale‐up efforts in this setting.

## 2 METHODS

### 2.1 Ethics statement

Ethical approval was granted by the Clinical Research Ethics Committee of the Cork Teaching Hospitals (CREC), CREC Review Reference Number: ECM 4 (y) 09/05/2023. Participants received study information and provided written informed consent.

### 2.2 Study design

A sequential explanatory mixed‐methods process evaluation^18^, ^19^ entitled ‘*Standardised Parenteral Nutrition for Preterm Infants: National Study of Current Use and Future Opportunities’* was conducted in two phases (Supporting Information: Figure S1). Phase 1 comprised a national survey of healthcare professionals (HCPs) and analysis of PN purchasing data to explore variation in implementation. Phase 2 involved a focus group with the National Implementation Team (NIT), a subgroup of the National PN Expert Group to contextualise survey findings, followed by interviews with HCPs. The NIT included clinicians who implemented the SPN system in their own site and supported national implementation, thus representing ‘key informants’. Integration of phases occurred through connection; sampling of Phase 2 participants and development of interview topic guide (Supporting Information: File S1) from Phase 1 data, and expansion, where Phase 2 was used to explain and elaborate on Phase 1 findings.^20^ The Consolidated Criteria for Reporting Qualitative Research (COREQ) informed reporting (Supporting Information).^21^

### 2.3 Study context

Ireland has 19 maternity‐based NUs organised as local (Level 1, *n* = 11), regional (Level 2, *n* = 4), and tertiary (Level 3, *n* = 4) centres. Preterm PN is used in nine units; all Level 2 and 3 units and in one of the Level 1 units.

### 2.4 Description of the SPN system and national scale‐up project

PremSmart (Preterm Standardised Multimodal and Responsive Transitional) SPN system^16^, ^17^, ^22^ comprises a two‐bag aqueous‐SPN suite (Supporting Information: Table S1) (Primene®, Baxter Healthcare Ltd, Thetford, UK); starter (PremSmart‐1, low electrolyte) used for the first 48 h after birth and a follow‐on (PremSmart‐2, maintenance electrolytes and trace elements), both used with SMOFlipid® with vitamins (Fresenius Kabi, Graz, Austria). The protocol guides prescribing of SPN volumes based on the infants EFV and day of life (DOL), providing target, minimum and maximum SPN volumes (Supporting Information: Table S2).^16^ It was developed through a multidisciplinary collaboration between Cork University Maternity Hospital and University College Cork, using nutrient modelling^17^ of prospectively collected macronutrient data, and implemented in Cork University Maternity Hospital in 2018. It was subsequently endorsed for national use, replacing two nutritionally inferior national SPN products with roll‐out led by the NIT and completed in 2021.^15^ A programme logic model,^23^ developed from the implementation documentation and an interview of a ‘key informant’ summarises the national project in Table 1. The implementation strategies employed were mapped to the Expert Recommendations for Implementing Change taxomy.^24^

**Table 1:** Logic model for preterm SPN system national scale‐up project.

Program target	Preterm infants <32 week gestation or <1.5 kg or other preterm infants that are not expected to receive adequate enteral intake (i.e., ≥75% of nutritional requirements) within approximately 3–5 days.	
Evidence‐base	Delivery of nutrients within internationally recommended ranges to support appropriate growth and development. Use of SPN in the majority of preterm infants, due to its improved nutrition delivery and enhanced patient safety from product formulation through to prescribing and administration when compared to IPN. Standardising care pathways for preterm PN to reduce interprovider variability and enhance consistency of care.	
Program content	The SPN system was developed by a multidisciplinary research team across Cork University Maternity Hospital and University College Cork. Following successful implementation in the development site in 2018, it was endorsed for national use. A national implementation team was established to lead the national rollout, completed in 2021.	
Objective	Improved nutritional care and short‐and long‐term growth and neurodevelopmental outcomes.	
Program architecture	The SPN system is embedded into the daily nutritional management of preterm infants. The initial implementation had a specified timeline in which formal efforts were employed and national funding was provided. This is a multi‐site and structured intervention. The SPN system is anticipated to have both downstream, i.e., frontline and upstream, i.e., organisation impacts.	
Resources	1. Provision of pocket‐sized versions of the SPN protocol and ready reckoners. 2. Online training sessions. 3. Training resources including presentations to support local education.	
Implementation strategies	1. Access new funding. 2. Build a coalition. 3. Facilitate relay of clinical data to providers. 4. Capture and share local knowledge. 5. Conduct educational meetings. 6. Develop educational materials. 7. Facilitation. 8. Identify and prepare champions.	9. Promote adaptability. 10. Inform local opinion leaders. 11. Involve executive boards. 12. Mandate change. 13. Promote network weaving. 14. Use advisory boards and workgroups. 15. Use train‐the‐trainer strategies. 16. Centralise technical assistance.
Output	1. Suite of 2 SPN formulations (starter and follow‐on). 2. SPN dosing protocol, updated following implementation based on user feedback. 3. Clinical data from two observational studies at development site.	
Short to medium term outcomes	1. Adoption, fidelity of use and sustainment of the SPN system. 2. Preterm infants in Ireland achieve standardised evidence‐based care for preterm PN including recommended nutrient intakes using predominantly SPN. 3. Improved preterm growth. 4. Reduced resource use in terms of product cost and staff time. 5. Reduction in use of higher risk IPN.	
Long‐term outcomes	1. Improved longer‐term health outcomes in preterm infants with associated cost savings. 2. National benchmarking and continuous quality improvement of preterm nutritional care.	

Abbreviations: IPN, individualised parenteral nutrition; PN, parenteral nutrition; SPN, standardised parenteral nutrition.

### 2.5 Sampling and recruitment

In Phase 1, all HCPs in the target population (estimated *n* = 572) were eligible for inclusion. For each NU, a designated contact distributed the survey via email and group messaging. In Phase 2 respondents were approached via email. All members of the NIT (*n* = 3) participated in a focus group. For interviews, survey respondents consenting to follow‐up were purposefully sampled to cover the spectrum of survey responses,^25^ selecting three NUs. These included the development site, which has demonstrated high fidelity to the protocol^16^ and a Level 2 and a Level 3 unit who reported different routine protocol adaptations. Snowball sampling included a consultant, doctor‐in‐training (DIT), nurse, dietitian and pharmacist from each NU.

### 2.6 Data collection

Definitions and measures for implementation outcomes, strategies, and determinants used in Phase 1 are summarised in Table 2.^24^, ^26^, ^27^, ^28^, ^29^, ^30^, ^31^ Sustainment was measured using the Provider Report of Sustainment Scale (PRESS).^30^ Implementation determinants were assessed using the Clinician Guideline Determinants Questionnaire (CGDQ), designed to measure factors influencing guideline adoption.^29^ Usability statements of the CGDQ were replaced with the Standard Usability Scale (SUS)^31^ to generate a baseline usability score. The survey (Supporting Information: File S2) was developed by consensus by the interdisciplinary research team and piloted for face validity and completion time. It was distributed and completed online using survey software (Qualtrics®) between October 2023 and March 2024. Qualitative data (Phase 2) was collected via Microsoft Teams® by SF; one interview was documented via notes due to connectivity issues. Sessions were video recorded, transcribed, verified and pseudonymised. The focus group lasted 63 min (April 2024); interviews lasted a median 41 min (interquartile range [IQR] = 11 min), (June 2024–July 2025).

**Table 2:** Phase 1 implementation construct definitions and measures.

Construct	Short description	Measurement
Implementation outcomes		
Adoption	Uptake of the SPN system including protocol at national, unit and individual level	National purchasing data: SPN (%) Unit level self‐reported implementation (%) Individual‐level self‐reported use (%)
Sustainment	Extent to which a recently implemented practice is maintained and institutionalised within a service setting's ongoing operations	Scoring of PRESS. 3 items, 5‐point Likert scale (0–4), averaged score; with higher scores indicating greater intervention sustainment
Fidelity	Degree to which an the intervention was delivered as intended in the original protocol	Self‐reported use of core components of the system's protocol and individual and unit level routine adaptations
Implementation strategies		
Training and education	Format and dose of education and training received	Self‐reported receipt of training or education
Point‐of‐care protocol	Availability of point‐of‐care SPN protocol	Unit and individual level self‐reported availability and use of protocol.
Availability of specialist staff	Availability of dietitian/pharmacist to support PN delivery	Unit level self‐reported availability of dietitian/pharmacist
Implementation determinants		
Attitudes, confidence, support from peers and organisation and evidence for SPN protocol	Contextual factors that influence implementation success are referred to as implementation determinants, often called barriers and facilitators	15 relevant items from the ‘Clinician Guideline Determinants Questionnaire; 7‐point Likert scale (1–7) with ‘not sure’ option (coded 0)
Usability of protocol	Usability of paper‐based SPN protocol	Scoring of SUS. SUS is a 10‐item scale yielding a 0–100 composite usability score with higher scores indicating greater perceived usability

Abbreviations: PRESS, Provider Report of Sustainment Scale; SPN, standardised parenteral nutrition; SUS, Standard Usability Scale.

### 2.7 Qualitative analysis

Thematic analysis was conducted using the framework method^32^ (Supporting Information: Figure S2). Coding was primarily deductive guided by the Consolidated Framework for Implementation Science Research (CFIR) constructs.^33^ Coding was undertaken iteratively, allowing refinement of codes and development of a bespoke project codebook to ensure all data were appropriately represented (Supporting Information: Table S3a–c).^34^ Interviews and the focus group were initially coded by SF, and seven interviews were independently coded by AF. NVivo (Release 1.7.2) was used for data analysis.

### 2.8 Reflexivity

Author, SF, a neonatal pharmacist with professional connections to participants but independent of the national implementation project, maintained a reflexive journal to explore how her positionality influenced the research. Co‐author AMB, a member of the former NIT was not involved in data analysis. Co‐authors CH and AF, researchers independent of the healthcare association delivering the intervention, acted as critical reviewers throughout.

### 2.9 Statistical analysis

Continuous variables are summarised using the mean (standard deviation, SD) and/or the median (interquartile range, IQR), and categorical variables using frequency (percentage). Fisher's exact test was used to compare categorical variables between groups. Continuous variables were compared using Mann–Whitney (two groups) or Kruskal–Wallis (≥3 groups) tests, with Dunn's test used for pairwise comparisons following a significant Kruskal–Wallis test. Associations between implementation determinants and background variables were analysed excluding ‘unsure’ responses. All tests were two‐sided, and a *p*‐value < 0.05 was considered statistically significant. IBM® SPSS Statistics v29 was used for the analysis.

## 3 RESULTS

### 3.1 Participants

In Phase 1, of 185 survey responses, 27 were excluded (13 did not provide consent, 13 completed ≤1 question and 1 was not using PN), leaving 158 respondents for analysis. The response rate was 27.6%, ranging from 72/412 (17.5%) for nurses to 12/12 (100%) for pharmacists, with 31/73 (42.5%) of DIT, 30/61 (49.2%) of consultants, including 24/40 (60%) of consultant neonatologists and 13/14 (92.9%) of dietitians. In Phase 2, there were 19 participants (one focus group (*n* = 3) and 16 interviews). The characteristics of the participants are described in Table 3.

**Table 3:** Characteristics of participants in Phases 1 & 2.

Characteristics	Phase 1 (*n* = 158^a^) *n* (%)	Phase 2 (*n* = 19) *n* (%)
Gender		
Male	29 (18.4)	5 (26.3)
Female	128 (81.0)	14 (73.7)
Prefer not to say	1 (0.6)	0 (0)
Age		
18–24 years	2 (1.3)	0 (0)
25–34 years	42 (26.6)	3 (15.8)
35–44 years	39 (24.7)	2 (10.5)
45–54 years	51 (32.3)	11 (57.9)
55–64 years	23 (14.6)	3 (15.8)
65 years or older	1 (0.6)	0 (0)
Professional role		
Nurse	72 (45.6)	4 (21)
Doctor in training	31 (19.6)	3 (15.8)
Consultant	30 (19.0)	4 (21)
Dietitian	13 (8.2)	5 (26.3)
Pharmacist	12 (7.6)	3 (15.8)
Years of experience in care of preterm infants		
Less than 1 year	10 (6.3)	0 (0)
1–5 years	37 (23.4)	2 (10.5)
6–10 years	27 (17.1)	2 (10.5)
11–15 years	17 (10.8)	2 (10.5)
16–20 years	16 (10.1)	2 (10.5)
Greater than 20 years	51 (32.3)	11 (57.9)
Unit level^b^	(*n* = 156)	
Level 1	25 (16.0)	0 (0)
Level 2	46 (29.5)	5 (26.3)
Level 3	85 (54.5)	14 (73.7)
Received training or education on how to use the SPN protocol	(*n* = 152)	Not captured
Yes	128 (84.2)	n/a
No	24 (15.8)	n/a

a Unless otherwise stated.

b Level 1 (local), Level 2 (regional) and Level 3 (tertiary).

### 3.2 Phase 1: Quantitative findings

#### 3.2.1 Implementation outcomes

##### 3.2.1.1 Adoption

The percentage of total preterm PN bags purchased as SPN increased nationally from 3662/6522 (56%) pre‐implementation (2017) to 4823/5074 (95%) in 2022, the year following national rollout,^15^ with site‐level SPN purchasing ranging from 89% to 100%. Survey respondents (*n* = 152), all reported that the SPN system had been introduced into their unit. Of 150 respondents who reported personal use, 114/150 (76%) reported ‘Always’ and 29/150 (19.3%) ‘Often’ using the protocol. There was no association between NU Level and reported personal protocol use (*p* = 0.659). Reported personal protocol use differed significantly by role (*p* = 0.043) with pharmacists less likely to answer ‘Always’ and more likely to answer ‘Often’ compared with other groups (Supporting Information: Figure S3).

##### 3.2.1.2 Sustainment

The median (IQR) PRESS score was 3.7 (3.3–4), (max score of 4). There was a significant difference in scores across unit levels (*p* = 0.002), with lower scores in Level 2 units compared to Level 1 (pairwise *p* = 0.016) and Level 3 (pairwise *p* < 0.001). Scores were higher among respondents who had received training (*p* < 0.001). Comparison between groups are presented in Supporting Information: Table S4.

##### 3.2.1.3 Fidelity

Routine unit level practices differing from the protocol (adaptations) were reported by 31/148 (20.9%), while 37/148 (25.0%) responded 'I don't know'. Adaptations did not vary by unit level (*p* = 0.310) but did by training status (*p* < 0.001). Untrained respondents were more likely to answer ‘I don't know' (59.1% vs. 19.0%) and less likely to answer ‘No' (22.7% vs. 59.5%) when asked whether adaptations occurred, compared with trained respondents. Twenty‐five respondents provided details of 54 adaptations across eight categories (Supporting Information: Figure S4). The most frequent adaptations were to the volume of BMF (20/54), routine use of the protocol in infants >32 week gestation/1.5 kg (7/54), and deviations from the recommended target SPN volumes (7/54).

#### 3.2.2 Implementation strategies

##### 3.2.2.1 Training and education

Training or education on the SPN system was received by 128/152 (84.2%) of respondents with no difference between NU level (*p* = 0.059) or professional role (*p* = 0.113). Of those who provided details, ‘On‐the‐job support from colleagues’ was the most frequently reported format (77/127, 60.6%), followed by local sessions, once‐off (75/127, 59.1%) or ongoing (54/127, 42.5%). Less than 30% of respondents (36/127, 28.3%), reported having the initial implementation training.

##### 3.2.2.2 Point‐of‐care protocol

All respondents (*n* = 152) had at least one format of protocol available, with 72/152 (47.4%) having both paper and electronic (static document created locally), 72/152 (47.4%) paper only and 8/152 (5.3%) electronic only.

##### 3.2.2.3 Availability of specialist staff

Availability of dietitians and/or pharmacists to support preterm PN use was reported by most respondents 148/156 (94.9%), and of those, 121/148 (81.8%) reported access to both a dietitian and a pharmacist, 15/148 (10.1%) to a dietitian only, and 12/148 (8.1%) to a pharmacist only.

#### 3.2.3 Implementation determinants

##### 3.2.3.1 Responses to clinician guideline determinants questionnaire

**Figure 1:**
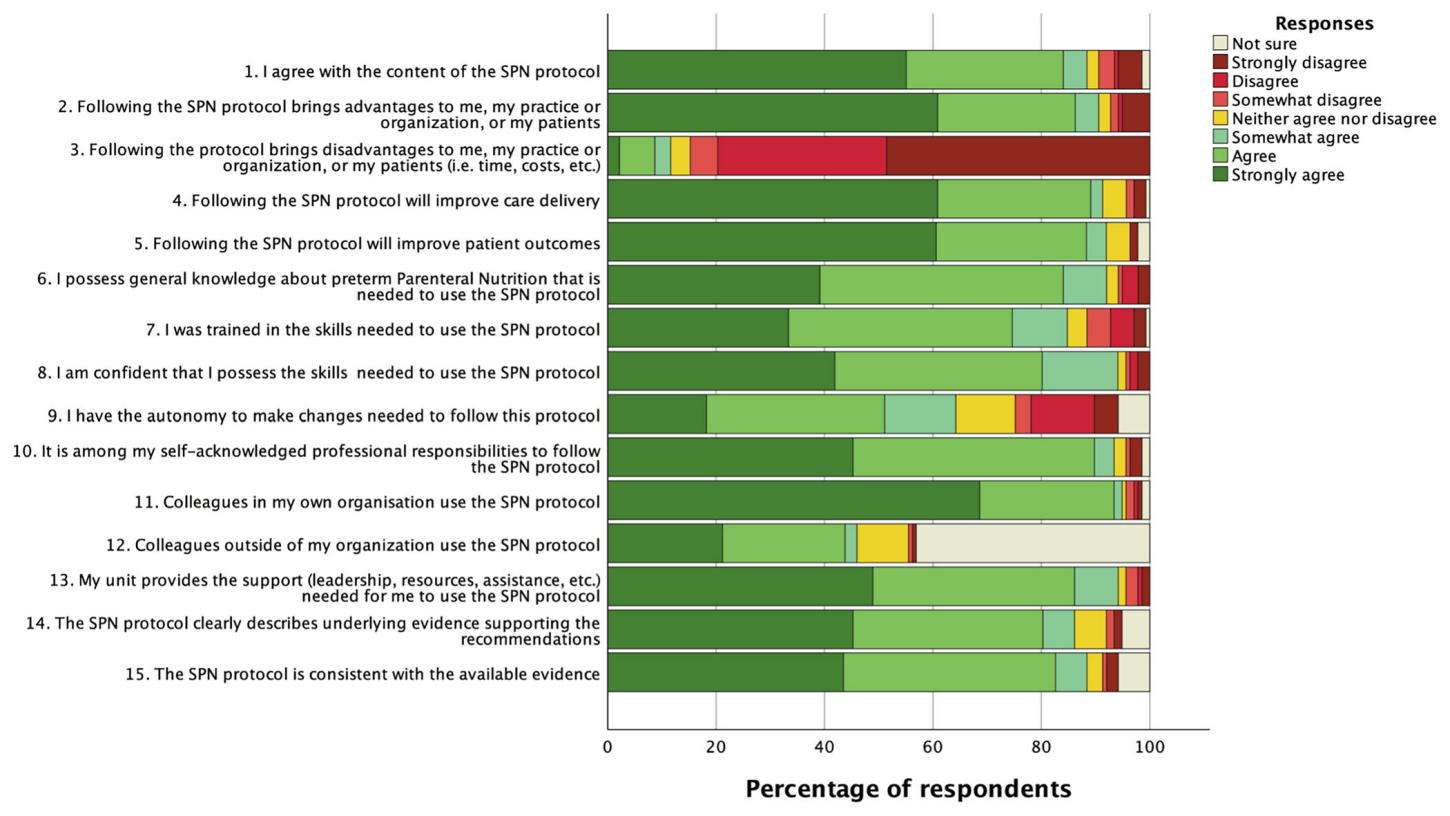
Healthcare Professionals responses to Likert statements for the Clinician Guideline Determinants Questionnaire. SPN standardised parenteral nutrition.

Responses to all 15 determinants are shown in Figure 1. Over 40%, 59/137 (43.1%) of respondents were uncertain whether colleagues outside their organisation used the SPN protocol. Greater years of experience (>10 years vs. 0–10 years) was associated with less favourable attitudes towards the use of the protocol and its evidence‐base (Supporting Information: Table S5). Training was associated with greater confidence in using the protocol and support from peers and the organisation in its use (Supporting Information: Table S5). NU level was associated with attitudes and confidence, with lower score from respondents from Level 2 units compared to Level 3 units (Supporting Information: Table S5). Differences by professional role were also observed, with nurses rating the protocol's advantage lower, and pharmacists their skills and confidence lower (Supporting Information: Table S5). Consultants reported higher autonomy to make protocol changes, and dietitians were more aware of its use outside of the organisation and more likely to view it as evidence‐based.

##### 3.2.3.2 Usability of the SPN protocol

SUS scores were calculated for 137 respondents, yielding a mean (SD) of 72.9 (16.2) and a median (IQR) of 75 (65–83.8), (max score of 100), equivalent to a ‘B’ grade and an adjective rating of ‘Good’. Scores were higher among DIT and dietitians than nurses, among staff with ≤ 10 years’ experience, in those with training and in Level 3 compared to Level 2 units (Supporting Information: Table S4).

### 3.3 Phase 2: Qualitative findings

**Figure 2:**
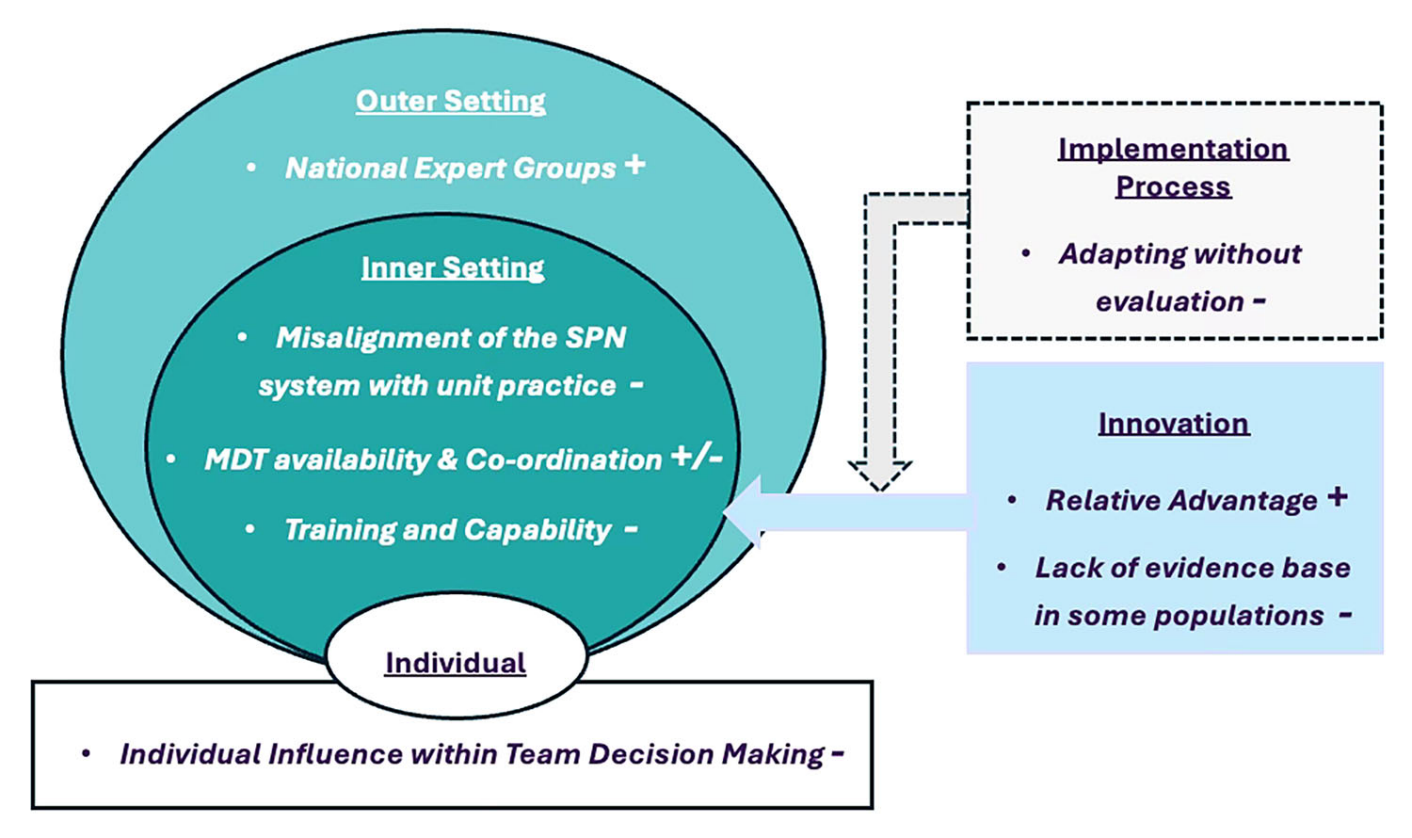
Key themes influencing national scale‐up of the preterm SPN system within the Consolidated Framework for Implementation Research (CFIR) domains. (adapted from the Centre for Implementation CFIR Toolbox). **−** Barrier/**+** Facilitator/**+/−** Facilitator or Barrier depending on the context. MDT, multidisciplinary team; SPN, standardised parenteral nutrition.

Quantitative findings were reviewed with the NIT to interpret patterns of high adoption alongside reported variations across unit levels and professional roles. Training and education gaps, reported protocol adaptations and differences in attitudes across unit acuity levels and professions shaped the focus of the subsequent interviews. Seven themes were developed to describe factors influencing scale‐up. Themes reflected various CFIR constructs, often spanning multiple domains and are presented with the domain it most closely aligned, illustrated in Figure 2. Additional illustrative quotes are presented in Table 4.

**Table 4:** Themes with illustrative quotations organised by CFIR domains.

CFIR domain	Theme	Illustrative quote
Innovation	Relative advantage of the SPN system and evidence‐base: Perception of the innovation and characteristics	“I think everyone on the whole welcomed it and they thought it was a great addition to have the standardised approach” (Pharmacist, ID08) “The way I would take it is this; if they lose it [SPN protocol] or anything happens, they ask me for more” (Dietitian, ID12) “I personally find it very straightforward because you know you have the enteral volume, you have your maximum SPN and I find it very helpful to have a minimum and max” (DIT ID10) “We're level 2. So you know the 28 week plus, so the SPN just seems to cover everything that they need” (Dietitian, ID07) “We might have to do some guideline for the tinies” (Consultant, ID09)
Outer setting	National expert & professional groups: Policy, governance and professional networks	“and if there is any new information that comes through from national, we are the quickest people to know about it and to kind of communicate it and to adopt it” (Consultant ID04) “I was able to revert back to Dietitians on Implementation Team. You know, with questions about particular babies and how I use the protocol in, in terms of the babies that didn't exactly fit the criteria” (Dietitian ID07) "We had a guideline [in other Level 2 site] that we used the Level 3 unit guidelines because we were working with the Level 3 unit, so it wasn't too difficult to implement I felt" (Nurse ID11)
Inner setting	Unit fit and user attitudes towards SPN system	“I think in our unit… one of the things was that it just didn't match up well with what we did for the late preterm” (Pharmacist ID08) “It doesn't tend to work well for us and in them bigger babies” (Dietitian ID07)
	MDT availability and co‐ordination	‘But unfortunately we're not always together at the one time. So I think that's lacking. I think that would make it better… I wouldn't see every baby on SPN” (Dietitian ID12). “if IPN needs to be ordered” (Pharmacist ID13) “Then I will ask the dietitian about her input and our dietitian is I think not daily” (DIT10, ID) “We were just thankful the dietitian was in that day because we were just looking for acetate. So we just hung an SPN 2 bag… and it kind of worked” (Pharmacist, ID08)) “We're all terrified of the day she [ANP] retires because she is the unit” (Pharmacist ID11) “So like we're as good as we are because of those two ladies [ANPs], I believe” (Nurse ID05)
	Training and capability – maintaining skills and expertise	“There is no sign off tool or anything like. There's no competency. It is just basically an education session” (Nurse ID11) “Like I can produce a timetable that says it is on, but can I be 100% certain that every single doctor showed up for it? I am not sure so” (Consultant ID01) “we're comfortable enough prescribing that on our own [SPN] if it's IPN, then that's done with heavy involvement from pharmacy and dietetics” (DIT ID15)
Individual	Individual influence within team decision making	“Certainly the hardest people to convince are the consultants because we are just a bit more cynical and tend to be older, so it potentially depends how influential the dietitian is in the unit. ultimately, if the consultant decides no, I don't think this is for me, it won't be ordered” (Consultant ID01) “And one of them…you know, just wouldn't listen to anyone, so that's a barrier” …“He has his own way around it. His own way of doing it” (Dietitian ID12) “So I know in ['Site X] for instance, there was a new neonatal dietitian on board, didn't have established relationships, you know because of being new. So that's going to have a different impact.”(Dietitian ID02)
Implementation process	Adapting without audit & evaluation	“it is being used, you know, not as it was intended …. what the percentage difference is or anything like that from a nutrition point of view, I wouldn't be able to tell” (Nurse ID11) *“*We did adapt, we adapted our feeding protocol” (Consultant ID09)

Abbreviations: ANP, advanced nurse practitioner; CFIR, Consolidated Framework for Implementation Research; DIT, doctor in training; IPN, individualised parenteral nutrition; MDT, multidisciplinary team; SPN, standardised parenteral nutrition; SPN 2, 'Follow on' SPN (PremSmart‐2).

#### 3.3.1 Innovation

##### 3.3.1.1 Relative advantage and evidence‐base

Participants predominantly perceived a clear advantage of the SPN system compared with the former individualised PN (IPN) model, citing benefits for patient safety, standardisation and nutritional optimisation. One Consultant (ID09) described “*the SPN is a huge improvement,…to uniformly have a good cocktail that would across the board be the standard…more safety based and optimising nutrition”*. The protocol design and its point‐of‐care format were also viewed as enhancing efficiency and simplicity of SPN ordering, with participants echoing the embedded use reported in the survey. At the Level 2 site, IPN ordering was eliminated entirely with a significant reduction reported at Level 3 sites. However, Level 3 sites highlighted the limited evidence supporting SPN use in specific populations, including surgical and the extreme preterm. As one Pharmacist (ID13) noted *“because we don't know the characteristics of those babies* (<23 week gestational age) *very well…I'm not sure how I feel about that”*.

#### 3.3.2 Outer setting

##### 3.3.2.1 Role of national expert and professional groups

During initial implementation, relationships between the NUs, the National PN Expert Group and the NIT were identified as a key facilitator. These relationships, often mediated by local champions, provided points of contact to troubleshoot implementation challenges. One Consultant (ID04) described this as “*Our dietitian was part of the national team…so she was kind of our champion… and she had the people who she could ask*”. However, the NIT was not funded beyond the roll‐out phase stage, and this discontinuity was reflected in participants' distinction between the initial implementation and business as usual. As one Dietitian (ID02) reflected “*After roll out it would have been nice to have some structured scheduled follow up for staff…to support ongoing use of the system*”.

#### 3.3.3 Inner setting

##### 3.3.3.1 Unit fit and user attitudes

The most significant barrier to protocol fidelity was the perceived lack of fit between the SPN system and pre‐existing nutrition policies. In the Level 2 unit, where PN was routinely provided to older preterm infants, the Dietitian (ID07) noted “*So say if a baby was on …80 mL per kilo of enteral feed day two, then you're only giving 8 mL of SPN 1 on Day 2 and 12 mls of lipid. You know, that's an awful waste for an SPN bag”*. This suggests the SPN system was perceived as less aligned with their local practices and may reflect the lower attitude scores reported in the survey among Level 2 sites. In contrast, the difference in volume of BMF in the Level 3 site had less impact on attitudes, being viewed by some as separate from SPN management. As one Consultant (ID09) explained “*I do think we follow the protocol. You know, not the fortification, it isn't part of the SPN protocol. That's the enteral feeding protocol*”.

##### 3.3.3.2 Multidisciplinary team availability and co‐ordination

All units had access to a pharmacist and a dietitian, however, there was significant variation in how these roles were co‐ordinated to support daily PN ordering. The development site had daily input, with the Consultant (ID14) noting *“Monday to Friday, there would be a pharmacist and a dietitian pretty much every day”*. In contrast other sites relied on reactive or more intermittent support. The presence of a dietitian and or a pharmacist during PN ordering supported protocol compliance providing “*checks and balances*” (Consultant, ID14), particularly during rotation of training doctors and the management of more complex cases. This, together with the support of nurses, was highly valued by other team members, as noted by one DIT (ID15): “*The nurses are quite good at saying look, we need to drop the PN*”. Specialist nurses and ANPs were identified as particularly influential figures on all sites. Variability in multidisciplinary team presence was attributed to staff turnover, recruitment challenges and competing demands on limited specialist time.

##### 3.3.3.3 Training and capability

Participants in all sites described challenges in maintaining formal training, with gaps across all professions and high reliance on on‐the‐job learning. Following introduction of the SPN system, the national neonatal PN e‐learning module became obsolete, increasing the challenge to deliver training. Reaching all staff, particularly nurses, was difficult as one Dietitian (ID07) noted *“the changeover of nurses has been massive on the unit…so it's hard to capture everyone”*. Training primarily targeted DIT, who typically prescribe PN and was limited to one or two educational sessions without competency assessment. Ensuring attendance was challenging, and sessions were limited by staff turnover and capacity. Unclear roles and responsibilities for training further hindered delivery and content was not tailored to professional roles. The transition from IPN to SPN reduced opportunities for staff to build expertise as the volume‐based SPN ordering decreased the need to actively understand and calculate nutrient intakes. DITs reported confidence using the SPN protocol but relied heavily on support from the dietitian and pharmacist for IPN orders.

#### 3.3.4 Individual

##### 3.3.4.1 Influence within team decision making

Decision‐making was described as traditionally hierarchical, with consultants' clinical perspective shaping practice including in areas such as BMF and routine use of PN in older preterm infants. This contributed to protocol adaptations consistent with survey findings. As one Consultant (ID01) noted about BMF “*people got scared after a few babies. So we*'*ve just been really, really careful*”. The ability of champions or other multidisciplinary team members to influence practice depended heavily on their interprofessional relationship with the Consultant group as a Dietitian (ID02) from the development site explained “*I had an established relationship with the Consultants, we would have worked on policies and changing things over the years. I think trust is a huge part of this*”. In contrast other sites described high levels of workforce turnover impacting these relationships.

#### 3.3.5 Implementation process

##### 3.3.5.1 Adapting without evaluation

Sites actively adapted both the protocol and local policies for better intervention fit within their unit. As one Consultant (ID09) described *“In the last, maybe 2 years, we cut down our PN to the late preterms…we only give it up to 33* ^*+*^ *6, we stopped giving it up to 36* ^*+*^ *6”*. Overtime, adaptations became routine practices, reflecting a sustained lack of fidelity to the SPN protocol. Concurrently, there was no evidence of audit or evaluation to assess the impact of these adaptations.

## 4 DISCUSSION

This mixed‐methods study provides a real‐world evaluation of the scale‐up a preterm SPN system, comprising SPN formulations and dosing protocol. Our findings demonstrate that scale‐up of an SPN intervention across multiple NUs is achievable. However, despite high reported adoption and embedded use, adaptations to the protocol were reported, highlighting the influence of contextual factors. Key enablers across the *Innovation*, and *Outer Setting* CFIR domains supported scale‐up. These included the relative advantage of the SPN system over the former IPN‐dominant model and strong professional networks between national groups and end users. In contrast, barriers to fidelity reflected an interaction between the *Inner setting* and *Individual* domains, where pre‐existing policies, hierarchical decision‐making, and workforce turnover limited the implementation of all SPN protocol components when the system extended beyond the development site. Key insights from this evaluation highlight the importance of defining and monitoring core components within the SPN intervention, recognising the influence of hierarchical decision making and multidisciplinary team structures on intervention fidelity, and planning for sustainment from the outset.Our study revealed broad acceptance of standardising care, consistent with recent evidence of drivers of change in neonatal practice,^14^ however, this acceptance had limits. On one site the inclusion of BMF at 80 mL/kg/d within the SPN intervention, a threshold defined during nutrient modelling,^17^ was not implemented. This reflected both a lack of clarity that BMF formed a core intervention component and a context‐driven adaptation in response to concerns regarding necrotising enterocolitis. Implementation strategies such as audit and feedback may help balance fidelity, ensuring core components are delivered as intended while supporting necessary adaptation to local context by providing feedback on intervention delivery and the nutritional impact of adaptations.^35^ For example, Johnson et al. combined nutritional audit with a Normalisation Process Theory approach to the planning and evaluation of nutrition guideline implementation to successfully improve nutrient intakes and growth in a single NU.^36^ Additionally, while we applied CFIR as a summative evaluation of implementation determinants, use prior to scale‐up may support prospective identification of implementation barriers.^37^

Another important insight from this evaluation was the influence of hierarchical decision making and the multidisciplinary team structure on how the system was implemented in practice. Survey and interview findings indicated that consultants perceived greater autonomy to modify the protocol, with other staff reporting less authority to challenge this. This dynamic reflects well described hierarchical norms in healthcare^38^ and contributes to tension between standardisation and professional autonomy.^39^, ^40^ In the development site, protocol fidelity was supported by the dietitian and pharmacist on the neonatal ward round. In contrast, the sites with protocol adaptations reported less routine multidisciplinary ward round presence. These dynamics were compounded by workforce turnover and difficulty recruiting specialist staff such as dietitians. Formalising neonatal nutrition support teams, in line with recommended standards for neonatal care,^41^ could institutionalise multidisciplinary team input at key decision points, support SPN intervention fidelity,^42^ and mitigate effects of staff turnover.

Finally, maintaining complex healthcare interventions over time is a recognised challenge^43^ and our findings highlight the need to consider sustainment of the SPN intervention from pre‐scale‐up.^9^ We identified several threats to sustainment, including training gaps and an evolving preterm population. Training was positively associated with key determinants of implementation and is considered an essential pre‐requisite for safe and effective neonatal PN practice.^3^, ^8^, ^10^, ^44^ Yet consistent with previous reports^6^ we identified high reliance on informal peer‐to‐peer training and limited use of structured curricula. Additionally, all sites reported challenges maintaining training due to workforce capacity constraints and unclear roles and responsibilities. Sustainment is further threatened by the increasing survival of extremely preterm infants, necessitating ongoing review of the evidence‐base underpinning the SPN system design. Initial implementation was enabled by strong connectivity between units and the NIT, but this team was not resourced beyond initial implementation. Although the SPN system is now embedded within national guidelines,^44^ sustainment will require continued investment.^9^ This should include infrastructure aligned with international consensus on optimising preterm care delivery,^3^, ^10^, ^41^ encompassing training, clinical audit and integration into health information systems to enable surveillance, benchmarking and quality improvement.

Limitations of this study include the low response rates to the survey which may introduce response bias. Other survey limitations include biases inherent in Likert scales,^45^ and the inability to analyse site‐level differences due to anonymity. As this was an exploratory study, no adjustment for multiple testing was conducted and findings should be interpreted as hypothesis‐generating.^46^ Strengths of this study include the sequential mixed‐methods design and engagement of key stakeholders to enable consideration of multiple perspectives and data triangulation, providing a comprehensive exploration of implementation. Scale‐up represents a new context outside of tightly controlled trials or well‐supported settings where interventions face new barriers such as differences in local resources, staff training or culture.^12^, ^35^ Our evaluation examines rollout across multiple units using CFIR, a framework recently advocated to strengthen the rigour of programme evaluation in nutrition practice.^37^ This facilitated identification of context‐specific barriers and facilitators that would not be routinely revealed by traditional clinical evaluation and provides a foundation to design implementation strategies to address these challenges. Future preterm PN research should consider hybrid effectiveness‐implementation designs that integrate process and outcome evaluation. Such designs enable simultaneous assessment of clinical effectiveness and implementation processes, clarifying how, why and under what conditions complex interventions such as PN achieve their effects to support the translation of evidence‐based nutrition practices into sustainable, system wide improvement.^47^

## 5 CONCLUSION

Despite consensus on the need for increased standardisation of preterm PN care including SPN formulations, implementation remains inconsistent internationally. This national study demonstrates scale‐up of an SPN intervention across multiple NUs is achievable, but in addition to SPN formulations and an SPN dosing protocol, system‐level infrastructure and ongoing monitoring are required. The findings will inform the development and costing of stakeholder‐informed tailored strategies to optimise sustainment and provides insights to guide future scale‐up efforts.

## CONFLICT OF INTEREST STATEMENT

The authors declare no conflict of interest.

## Supporting information

Suppl_Figure 1.

Suppl_Figure_S2.

Suppl_Figure_S3.

Suppl_Figure_S4.

Suppl_File_S1.

Suppl_File_S2.

Suppl_Table S1.

Suppl_Table S2.

Suppl_Table_S3.

Suppl_Table_S4.

Suppl_Table_S5.
