# Supplementary figures and images for: Barriers and facilitators to the national scale‐up of a preterm standardised parenteral nutrition system: A mixed‐methods evaluation
Source: JPGN Rep. 2026 Jul 31:10.1002/jpr3.70213. Online ahead of print. doi: 10.1002/jpr3.70213 (PMC13425788; doi:10.1002/jpr3.70213)

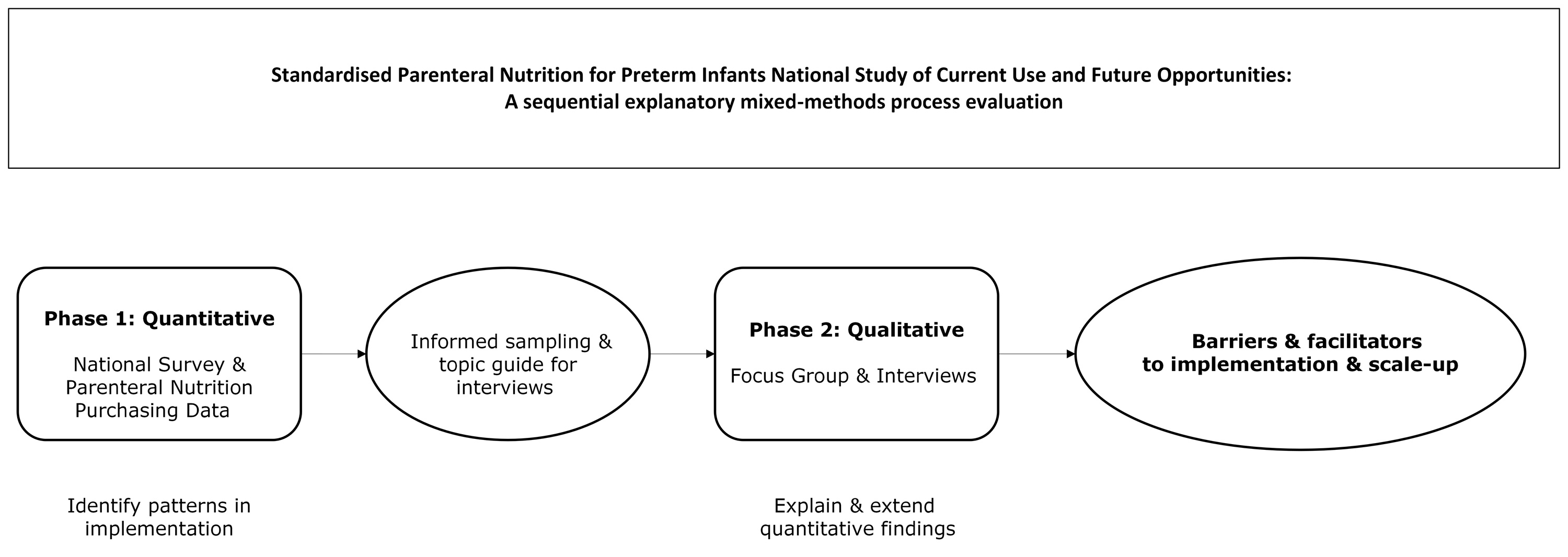

Supplement: Supplementary file 1 — Suppl_Figure 1. [file JPR3-9999-0-s011.jpg]

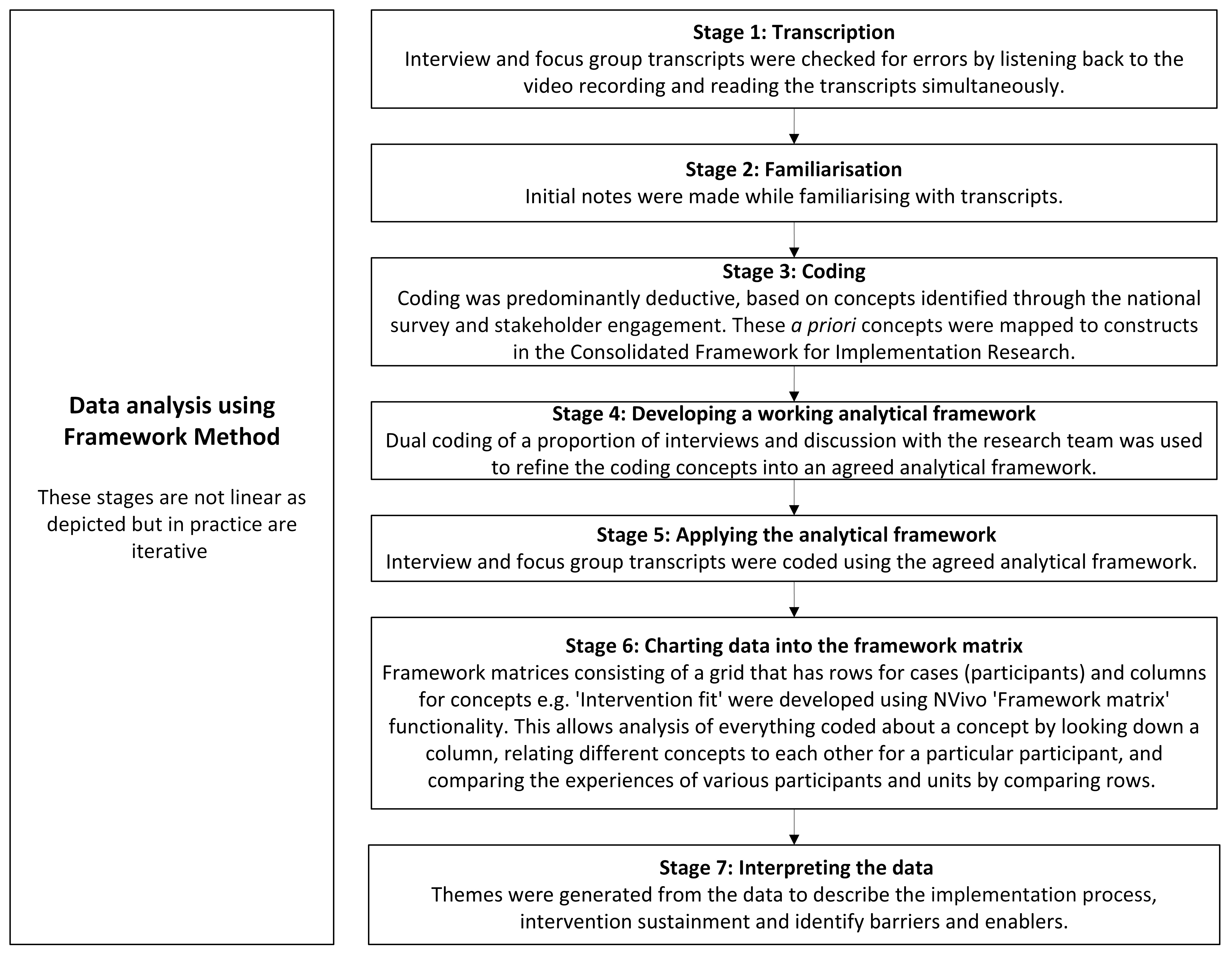

Supplement: Supplementary file 2 — Suppl_Figure_S2. [file JPR3-9999-0-s004.jpg]

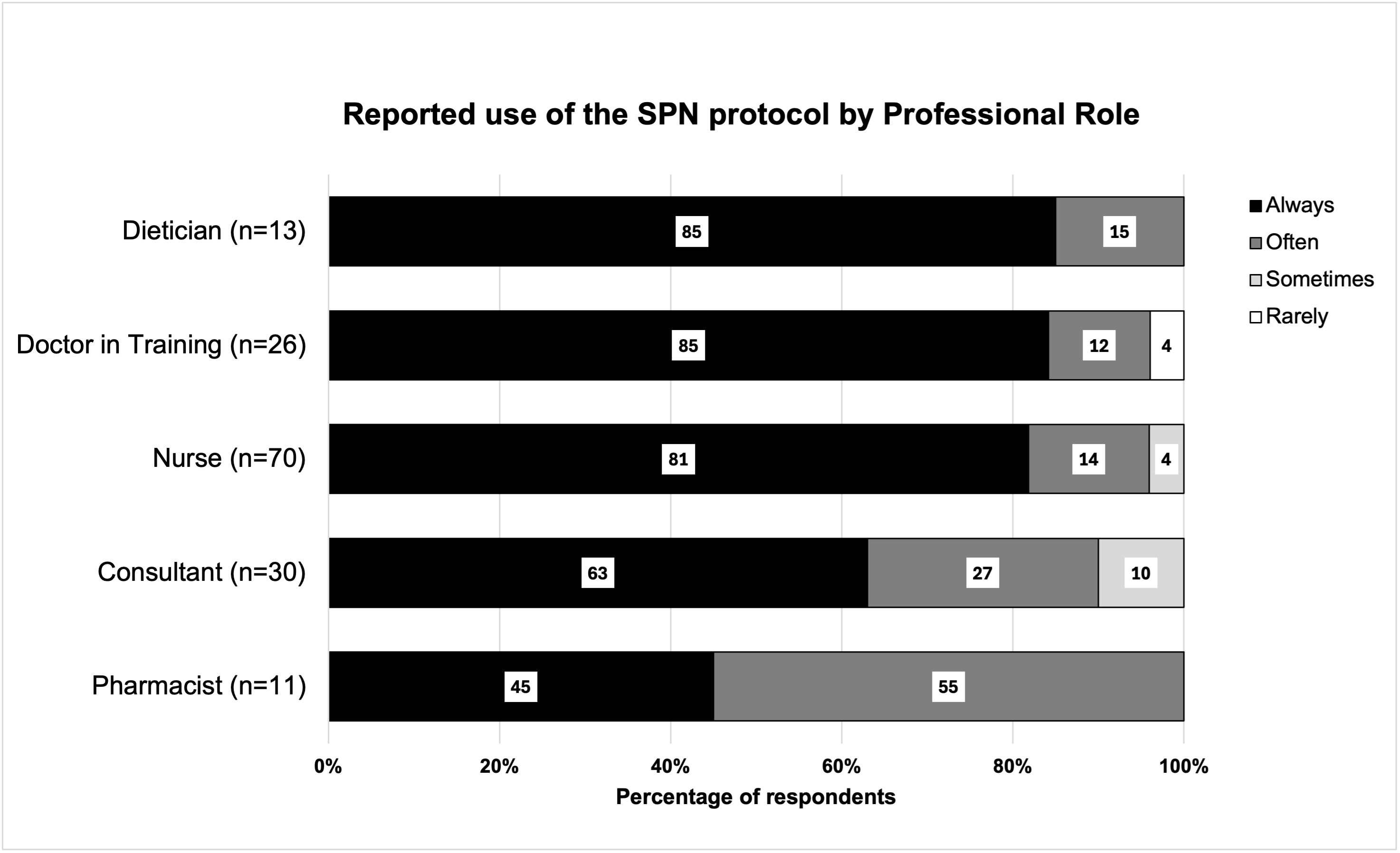

Supplement: Supplementary file 3 — Suppl_Figure_S3. [file JPR3-9999-0-s006.png]

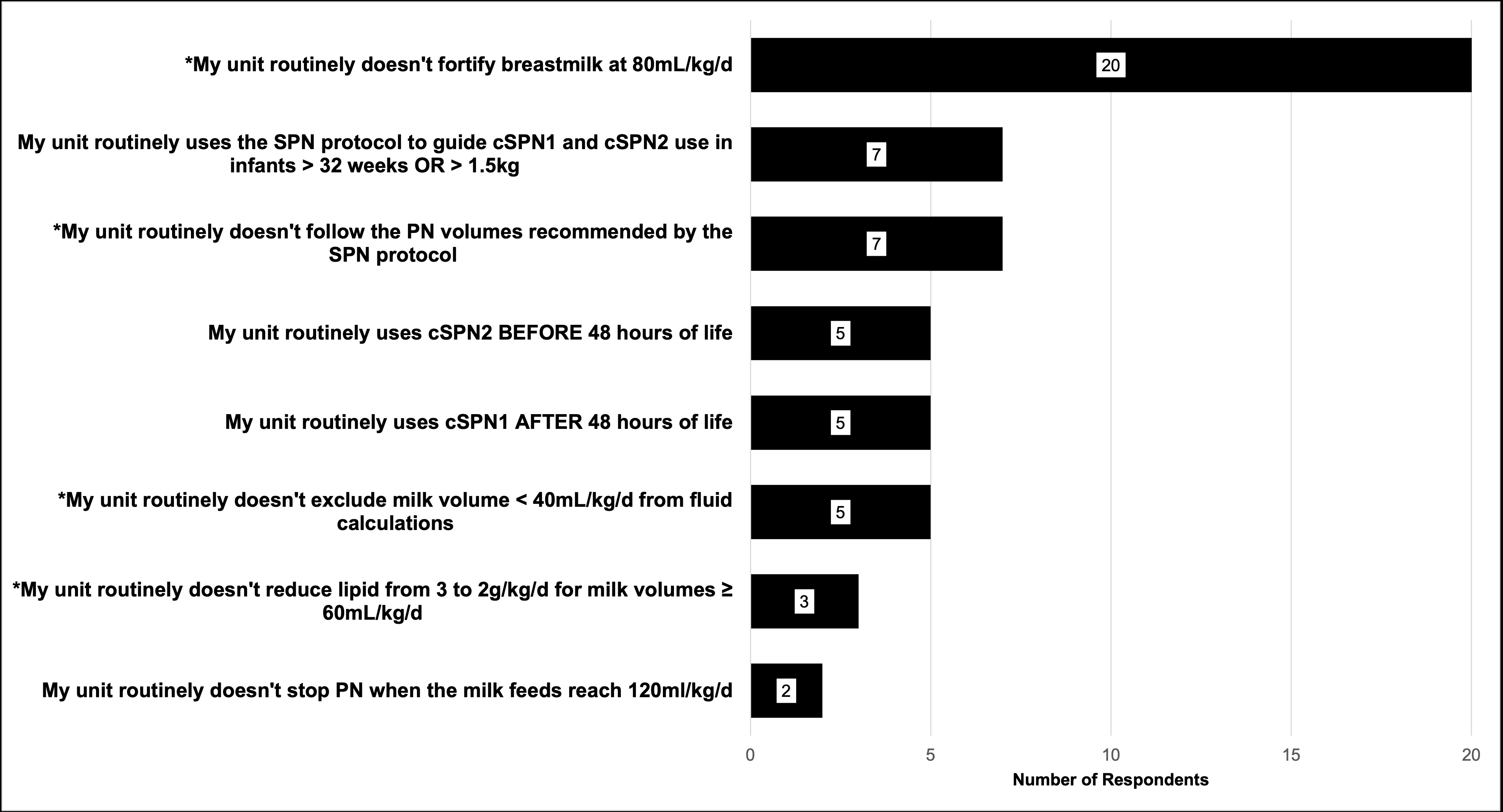

Supplement: Supplementary file 4 — Suppl_Figure_S4. [file JPR3-9999-0-s005.jpg]
