# Supplementary material for: Barriers and facilitators to the national scale‐up of a preterm standardised parenteral nutrition system: A mixed‐methods evaluation
Source: JPGN Rep. 2026 Jul 31:10.1002/jpr3.70213. Online ahead of print. doi: 10.1002/jpr3.70213 (PMC13425788; doi:10.1002/jpr3.70213)
Supplement: Supplementary file 5 — Suppl_File_S1. [file JPR3-9999-0-s007.docx]

**SPN Study Interview Topic Guide**

1. Can you tell me about your current role, recent role in the neonatal unit

Help me understand your role, tell me about your role

- Title, Main responsibilities, Number of years of experience, Age range

1. Can you tell me about your involvement in the use of Parenteral Nutrition
2. Can you talk me through how parenteral nutrition is ordered, – e.g. ward round, who is present, roles and responsibilities
3. What are your general thoughts about the SPN system? Unintended consequences.
4. If applicable – What was your experience of introducing the SPN system into your unit?
   1. What were the main challenges of introducing the SPN system into your unit?
   2. Was it possible to resolve those challenges over time?
   3. What factors helped you or enabled you to implement the programme – e.g. champions
5. Was the SPN system adapted to fit in with your unit – workflow, dependent policies such as fluid policies, Enteral feeding policy? – Were there policies and procedures in place that impacted the implementation/use of the SPN protocol.
6. Are there variations in the way different staff use the SPN protocol?
7. If applicable, e.g. Doctors in Training – have you used the SPN system/ other SPN in another site – are there variations in the way this was used.
8. Training – how does you unit maintain SPN training levels –
   1. What are the challenges?
   2. Doctors in Training - What is your experience of training/on the job support.
   3. What additional training or educational resources, if any would
9. Audit – are you aware whether audit and feedback of SPN system occurring. Do you think it is feasible to undertake routine nutritional audit.
10. Is there anything about the SPN system or the way it is used that you would change going forward.

Is there anything else you didn’t mention that you would like to add?
