# Supplementary material for: Barriers and facilitators to the national scale‐up of a preterm standardised parenteral nutrition system: A mixed‐methods evaluation
Source: JPGN Rep. 2026 Jul 31:10.1002/jpr3.70213. Online ahead of print. doi: 10.1002/jpr3.70213 (PMC13425788; doi:10.1002/jpr3.70213)
Supplement: Supplementary file 6 — Suppl_File_S2. [file JPR3-9999-0-s008.docx]

**Standardised Parenteral Nutrition for Preterm Infants - National Study of Current Use and Future Opportunities: Healthcare Professional Survey**

**Section 1: This section asks about you**

Q1.1 What is your gender?

- Male
- Female
- Non-binary
- Prefer not to say
- Another option not listed (please specify below) __________________________________________________

Q1.2 What is your age group, please select the appropriate age category.

- 18-24 years
- 25-34 years
- 35-44 years
- 45-54 years
- 55-64 years
- 65 years or older

Q1.3 What is your current role?

- Neonatal nurse
- Clinical Nurse Manager
- Advanced Nurse Practitioner
- Consultant Neonatologist
- Consultant Paediatrician
- Neonatal/Paediatric Doctor in Training
- Dietitian
- Pharmacist
- Other (please specify below) __________________________________________________

Q1.4 How many years' experience do you have in the care of preterm infants?

- Less than 1 year
- 1 - 5 years
- 6-10 years
- 11-15 years
- 16-20 years
- Greater than 20 years

Q1.5 In what way are you currently involved with preterm Parenteral Nutrition (PN)? (Select all that apply)

- Prescribing
- Advising
- Checking
- Administration
- Other please specify below __________________________________________________
- I currently am not involved with preterm PN (exit survey if selected)

Q1.6 In the past 3 months, on average, how often were you involved in the use of preterm PN?

- Daily (at least once a day)
- Weekly (at least once a week)
- Monthly (at least once a month)
- Occasional use (less than once a month)
- Never

End of Block: About you

Start of Block: Your Neonatal Unit

**Section 2: This section asks about the neonatal unit you work in and its use of PN and the SPN protocol.**

________________________________________________________________

Q2.1 Please select the type of neonatal unit you work in?

- Level 1 Neonatal Unit
- Level 2 Neonatal Unit
- Level 3 Neonatal Unit
- Other (please specify below) __________________________________________________

Q2.2 Does your neonatal unit care for infants who have complex cardiac conditions or are post-surgical i.e., gastrointestinal surgery? 

- Yes
- No

Q2.3 Please select the allied health professionals available to support the use (prescribing OR advising OR checking OR administration) of PN in your unit (please select all that apply)

- None
- Dietitian
- Pharmacist

Q2.4 Does your unit have an Enteral Feed Advancement Guideline in place?

- Yes
- No

Q2.5 How often is the Enteral Feed Advancement Guideline currently followed in your unit?

- Always
- Often
- Sometimes
- Rarely
- Never

Q2.6 Has the national SPN system (cSPN1, cSPN2 and protocol) been introduced into your neonatal unit? 

- Yes
- No

Q2.7 What format(s) of the SPN protocol is available to use in your unit? (please select all that apply)

- Paper: Pocket sized i.e., on lanyard for personal use
- Paper: A4 protocol for personal use
- Paper: A4 protocol centrally accessed in unit
- Electronic: Protocol centrally accessed on desktop or tablet
- We don't use the SPN protocol in our unit
- Other (please specify below) __________________________________________________

Q 2.8 The following asks about the use of the SPN protocol to guide cSPN1 and cSPN2 product use in your unit? Please indicate the extent to which you agree with the following items.

|  | Not at all | To a slight extent | To a moderate extent | To a great extent | To a very great extent |
| --- | --- | --- | --- | --- | --- |
| Staff use the SPN protocol as much as possible when appropriate |  |  |  |  |  |
| Staff continue to use the SPN protocol throughout changing circumstances |  |  |  |  |  |
| Using the SPN protocol is a routine part of our practice |  |  |  |  |  |

Q2.9 For what reason(s) is the SPN protocol not in use/not consistently used in your unit?

________________________________________________________________

________________________________________________________________

________________________________________________________________

________________________________________________________________

End of Block: Your Neonatal Unit

Start of Block: Personal use

**Section 3: This section asks about your individual use of the SPN protocol**

Q3.1 Have you had any training or education on how to use the SPN protocol?

- Yes
- No

Q3.2 If yes to above - through which of the options below? (please select all that apply)

- Online live training as part of the national rollout (once off)
- Local training or education (once off)
- Local training or education (ongoing i.e., more than a single session)
- On the job support from colleagues
- Other (please specify below) __________________________________________________

Q3.3 What is your current use of the SPN protocol when you are prescribing OR advising OR checking OR administering cSPN1 & cSPN2 products?

- Always
- Often
- Sometimes
- Rarely
- Never

Q3.4 Do you intend to use the SPN protocol in the future?

- Definitely won't
- Probably won't
- Unsure
- Probably will
- Definitely will

| Page Break |  |
| --- | --- |

Q3.5 What format(s) of the SPN protocol did or do you use? (please select all that apply)

- Paper: Pocket sized i.e., on lanyard for personal use
- Paper: A4 protocol for personal use
- Paper: A4 protocol centrally accessed in unit
- Electronic: Protocol centrally accessed on desktop or tablet
- I don't use the SPN protocol
- Other (please specify below) __________________________________________________

Q3.6 If you don't always/often use the SPN protocol, please describe reasons why?

________________________________________________________________

End of Block: Personal use

Start of Block: Adaptions

**Section 4:  This section asks about routine neonatal unit level and personal practices that differ from the SPN protocol recommendations.**

________________________________________________________________

Q4.1 In your unit are there any routine practices that differ from the SPN protocol recommendations e.g., timing of breastmilk fortification?

- No
- Yes
- I don't know

Q4.1b Please select any routine practices in your unit that differ from the SPN protocol recommendations (select all that apply)

- My unit routinely doesn't fortify breastmilk at 80mL/kg/d (Please explain reason why below) __________________________________________________
- My unit routinely doesn't exclude milk volume < 40mL/kg/d from fluid calculations (Please explain reason why below) __________________________________________________
- My unit routinely doesn't reduce lipid from 3 to 2g/kg/d for milk volumes ≥ 60mL/kg/d (Please explain reason why below) __________________________________________________
- My unit routinely uses cSPN1 AFTER 48 hours of life (Please explain reason why below) __________________________________________________
- My unit routinely uses cSPN2 BEFORE 48 hours of life (Please explain reason why below) __________________________________________________
- My unit routinely doesn't follow the PN volumes recommended by the SPN protocol (Please explain reason why below) __________________________________________________
- My unit routinely uses the SPN protocol to guide cSPN1 and cSPN2 use in infants > 32 weeks OR > 1.5kg (Please explain reason why below) __________________________________________________
- My unit routinely doesn't stop PN when the milk feeds reach 120mls/kg/d (Please explain reason why below) __________________________________________________
- Other (please specify and explain reason below) __________________________________________________

Q4.2 Do you have any routine personal practices when using the SPN protocol that differ to your unit's practices above? e.g., timing of breastmilk fortification?

- Not applicable to my current role
- No
- Yes (please specify below) __________________________________________________

Q4.3 Do you have any routine personal practices that differ from the SPN protocol recommendations e.g., timing of breastmilk fortification?

- Not applicable to my current role
- No
- Yes __________________________________________________

| Page Break |  |
| --- | --- |

Q4.3b (if yes to above) Please select any routine personal practices that differ to the SPN recommendations? (please select all that apply)

- I routinely don't fortify breastmilk at 80mL/kg/d (Please explain reason why below) __________________________________________________
- I routinely don't exclude milk volume < 40mL/kg/d from fluid calculations (Please explain reason why below) __________________________________________________
- I routinely don't reduce lipid from 3 to 2g/kg/d for milk volumes >/=60mL/kg/d (Please explain reason why below) __________________________________________________
- I routinely use cSPN 1 AFTER 48 hours of life (Please explain reason why below) __________________________________________________
- I routinely use cSPN 2 BEFORE 48 hours of life (Please explain reason why below) __________________________________________________
- I routinely don't follow the PN volumes recommended by the SPN protocol (Please explain reason why below) __________________________________________________
- I routinely use the SPN protocol to guide cSPN1 and cSPN2 use in infants > 32 weeks OR > 1.5kg __________________________________________________
- I routinely don't stop PN when the milk feeds reach 120mL/kg/d (Please explain reason why below) __________________________________________________
- Other (please specify below) __________________________________________________

End of Block: Adaptions

Start of Block: Determinants

**Section 5: This section asks about determinants of SPN protocol use**

________________________________________________________________

Q5.1 Please rate you level of agreement with the following statements

|  | Strongly disagree | Disagree | Somewhat disagree | Neither agree nor disagree | Somewhat agree | Agree | Strongly agree | Not sure |
| --- | --- | --- | --- | --- | --- | --- | --- | --- |
| I agree with the content of the SPN protocol |  |  |  |  |  |  |  |  |
| Following the SPN protocol brings advantages to me, my practice or organization, or my patients (i.e. supports communication and decision-making, etc.) |  |  |  |  |  |  |  |  |
| Following the protocol brings disadvantages to me, my practice or organization, or my patients (i.e., time, costs, etc.) |  |  |  |  |  |  |  |  |
| I possess general knowledge about preterm Parenteral Nutrition that is needed to use the SPN protocol |  |  |  |  |  |  |  |  |
| I was trained in the skills needed to use the SPN protocol |  |  |  |  |  |  |  |  |
| I am confident that I possess the skills needed to use the SPN protocol |  |  |  |  |  |  |  |  |
| Following the SPN protocol will improve care delivery |  |  |  |  |  |  |  |  |
| Following the SPN protocol will improve patient outcomes |  |  |  |  |  |  |  |  |
| The SPN protocol clearly describes underlying evidence supporting the recommendations (i.e., clear references) |  |  |  |  |  |  |  |  |
| The SPN protocol is consistent with the available evidence (i.e.national or international guidelines) |  |  |  |  |  |  |  |  |
| Colleagues in my own organisation use the SPN protocol |  |  |  |  |  |  |  |  |
| Colleagues outside of my organization use the SPN protocol |  |  |  |  |  |  |  |  |
| I have the autonomy to make changes needed to follow this protocol |  |  |  |  |  |  |  |  |
| It is among my self-acknowledged professional responsibilities to follow the SPN protocol |  |  |  |  |  |  |  |  |
| My unit provides the support (leadership, resources, assistance, etc.) needed for me to use the SPN protocol |  |  |  |  |  |  |  |  |
|  |  |  |  |  |  |  |  |  |
|  |  |  |  |  |  |  |  |  |
|  |  |  |  |  |  |  |  |  |

Q5.2 Who expects you to use the procedures, actions or activities recommended by the SPN protocol? (please select all

that apply)

- Patients
- Colleagues
- Managers or executives in my organisation
- Government
- Professional Society e.g., HSE Parenteral Nutrition Expert Group
- Other (please specify below) __________________________________________________

Q5.3 With respect to the 'USABILITY" of the SPN protocol, please rate your level of agreement with the following statements.

Please record your immediate response to each statement, rather than thinking about it for a long time. All items should be checked. If you feel that you cannot respond to a particular item, please mark the centre point of the scale.

|  | Strongly disagree | Disagree | Neither agree nor disagree | Agree | Strongly agree |
| --- | --- | --- | --- | --- | --- |
| I like to use the SPN protocol frequently |  |  |  |  |  |
| I find SPN protocol unnecessarily complex |  |  |  |  |  |
| I think the SPN protocol is easy to use |  |  |  |  |  |
| I need the support of an expert to be able to use the SPN protocol |  |  |  |  |  |
| I find the various components of the SPN protocol are well integrated. |  |  |  |  |  |
| I think there is too much inconsistency in the SPN protocol |  |  |  |  |  |
| I would imagine that most people would learn to use this protocol very quickly |  |  |  |  |  |
| I find the SPN protocol very cumbersome to use |  |  |  |  |  |
| I feel very confident using the SPN protocol |  |  |  |  |  |
| I needed to learn a lot of things (about the SPN protocol) before I could get going with the SPN protocol |  |  |  |  |  |

End of Block: Determinants

Start of Block: Digital version

**Section 6: This section will ask about the features of a future digital version of the SPN protocol that would be important to you.**

Q6.1 Below are possible formats of the SPN protocol. Please rank in order of preference (with 1 your most preferred).

______ Mobile (smartphone) application

______ Electronic version (software) on desk-top/laptop computer

______ Electronic version on a web site

______ Integrated into the electronic health record workflow

______ Print copy

______ Other (please specify below)

Q6.2 Below are possible features of a digital SPN protocol. If the SPN protocol becomes digital, what features would be important to you? (select all that apply)

- Speed of completing workflow
- Information for decision making presented at the time needed
- Fits into the workflow
- Easy to use
- Provides clinical decision support
- Clear why recommendations are being made
- Evidence based and up-to-date
- Minimal training required
- Minimal data entry
- Collects data e.g. for audit or measure outcomes
- Incorporates Enteral Feeding Advancement Guidelines
- Other (please specify) __________________________________________________

Q6.3 What one feature of a digital SPN protocol would be most important to you?

- Speed of completing workflow
- Information for decision making presented at the time needed
- Fits into the workflow
- Easy to use
- Provides clinical decision support
- Clear why recommendations are being made
- Evidence based and up-to-date
- Minimal training required
- Minimal data entry
- Collects data e.g. for audit or measure outcomes
- Incorporates Enteral Feeding Advancement Guidelines
- Other (please specify) __________________________________________________

Q6.4 Please indicate your preferred education and training formats (select all that apply)

- One to one support in clinic/practice area
- Face to face - in classroom
- Live online (virtual) training during work time
- Live online (virtual) training in my own time
- Online recording of training viewed during work time
- Online recording of training viewed in my own time
- Other (please specify below) __________________________________________________

End of Block: Digital version

Start of Block: Comments

**Section 7: Additional information
This section gives you opportunity to add any additional information and comments**

Please write any comments you want to make here. These could explain your answers in more detail or add any other information about the SPN protocol including barriers or facilitators to use or ideas for the future digital version. 

________________________________________________________________

________________________________________________________________

________________________________________________________________

________________________________________________________________

________________________________________________________________

End of Block: Comments

Start of Block: Block 4

You have reached the end of the survey. Thank you for taking the time to complete this survey.
We also want to talk to a selection of healthcare professionals in more detail about their experience of the SPN system and its usability in the current format through interviews. If you would be happy for us to contact you with more details, please indicate here and enter your name and email address below. Your responses to this survey will remain anonymous even if you provide your contact details. We will not use your email address for any purpose other than contacting you with more information about this study.

If you have any queries about this research, please contact Sarah Fenton at sarahfenton@umail.ucc.ie, Dr Ann-Marie Brennan at ann-marie.brennan@ucc.ie or Dr Brendan Murphy at brendan.murphy@ucc.ie. If you have any concerns about this research, you may contact your line manager or General Practitioner for support.

I am happy to be contacted, if selected, with more information about taking part in an interview.

- Yes, I am happy to be contacted if I am selected for interview.
- No, I would rather not be contacted about an interview

So we can contact you if you are selected for interview, please enter your email address here

________________________________________________________________

End of Block: Block 4
