# Supplementary material for: Barriers and facilitators to the national scale‐up of a preterm standardised parenteral nutrition system: A mixed‐methods evaluation
Source: JPGN Rep. 2026 Jul 31:10.1002/jpr3.70213. Online ahead of print. doi: 10.1002/jpr3.70213 (PMC13425788; doi:10.1002/jpr3.70213)
Supplement: Supplementary file 7 — Suppl_Table S1. [file JPR3-9999-0-s003.docx]

**Supplementary Table 1.** Composition of PremSmart Aqueous-Standardised Parenteral Nutrition Formulations

| **Per 100 mL** | **PremSmart-1** | **PremSmart-2** |
| --- | --- | --- |
| Energy, kcal | 54 | 51 |
| Amino acid, g | 3.9 | 3.3 |
| Glucose, g | 9.5 | 9.4 |
| Na, mmol | 1.0 | 3.0 |
| K, mmol | 1.0 | 1.4 |
| Ca, mmol | 1.0 | 1.4 |
| PO_4_, mmol | 1.0 | 1.5 |
| Mg, mmol | 0.1 | 0.2 |
| Acetate, mmol | 0.5 | 1.4 |
| Zn, ug | 327 | 325 |
| Junyelt, ml | 0 | 0.8 |
| Osmolarity, mOsmol/L | 900 | 900 |
