# Supplementary material for: Barriers and facilitators to the national scale‐up of a preterm standardised parenteral nutrition system: A mixed‐methods evaluation
Source: JPGN Rep. 2026 Jul 31:10.1002/jpr3.70213. Online ahead of print. doi: 10.1002/jpr3.70213 (PMC13425788; doi:10.1002/jpr3.70213)
Supplement: Supplementary file 8 — Suppl_Table S2. [file JPR3-9999-0-s010.docx]

**Supplementary Table 2.** The PremSmart enteral-feed-volume-based protocol with target macronutrient and energy intakes and ranges.

| Enteral Feed  Volume  mL/kg/day | | Target-Max SPN Volume  mL/kg/day | | |  | Amino Acid  g/kg/day | |  | Glucose  g/kg/day | |  | Lipid  g/kg/day | |  | Energy  g/kg/day | |
| --- | --- | --- | --- | --- | --- | --- | --- | --- | --- | --- | --- | --- | --- | --- | --- | --- |
|  |  | **Aqueous**^a^ | **Lipid**^b^ | **Total** |  | **Target Range**^c^ | **Target Intake**  *(PN+EN)* |  | **Target Range** | **Target Intake**  *(PN+EN)* |  | **Target Range** | **Target Intake**  *(PN+EN)* |  | **Target Range** | **Target Intake**  *(PN+EN)* |
| <40 | *DOL 1* | 65-65 | 6-12 | 71-77 |  | 1.5-2.5 | 2.5  *(2.5+0)* |  | 5.8-7.8 | 6.2  *(6.2+0)* |  | 1-2 | 1  *(1+0)* |  | 40-55 | 45  *(45+0)* |
|  | *DOL 2* | 80-90 | 12-18 | 92-108 |  | 2.5-3.5 | 3.1  *(3.1+0)* |  | 7-9 | 7.6  *(7.6+0)* |  | 2-3 | 2  *(2+0)* |  | 55-75 | 63  *(63+0)* |
|  | *DOL 3* | 95-120 | 18-14 | 113-144 |  | 3-4 | 3.1  *(3.1+0)* |  | 8.5-10.5 | 8.9  *(8.9+0)* |  | 3-4 | 3  *(3+0)* |  | 75-85 | 78  *(78+0)* |
|  | *DOL 4* | 105-120 | 18-24 | 123-144 |  | 3.5-4 | 3.5  *(3.5+0)* |  | 9.7-15 | 10  *(10+0)* |  | 3-4 | 3  *(3+0)* |  | 90-115^d^ | 85  *(85+0)* |
| 40 | | 95-100 | 18 | 153-158 |  | 3.5-4 | 3.8  *(3.1+0.7e)* |  | 9.7-15 | 11.7  *(8.9+2.8)* |  | 3-4^e^ | 4.5  *(3+1.5)* |  | 90-115 | 105  (78+27) |
| 50 | | 85-95 | 18 | 153-163 |  | 3.5-4 | 3.7  *(2.8+0.9e)* |  | 9.7-15 | 11.5  *(8+3.5)* |  | 3-4 | 4.8  *(3+1.8)* |  | 90-115 | 107  (73+34) |
| 60 | | 80-90 | 12 | 152-162 |  | 3.5-4 | 3.6  *(2.6+1e)* |  | 9.7-15 | 11.6  *(7.5+4.1)* |  | 3-4 | 4.2  *(2+2.2)* |  | 90-115 | 101  (61+40) |
| 70 | | 70-85 | 12 | 152-167 |  | 3.5-4 | 3.5  *(2.3+1.2e)* |  | 9.7-15 | 11.5  *(6.6+4.9)* |  | 3-4 | 4.6  *(2+2.6)* |  | 90-115 | 103  (56+47) |
| 80 | *FBM* | 60-75 | 12 | 152-167 |  | 3.5-4.5 | 4.1  *(1.8e+2.3)* |  | 11.6-13.2 | 12.2  *(5.6+6.6)* |  | 4.8-6.6 | 5.5  *(2+3.5)* |  | 110-130 | 117  (50+67) |
| 90 | *FBM* | 50-65 | 12 | 152-167 |  | 3.5-4.5 | 4.2  *(1.5e+2.7)* |  | 11.6-13.2 | 12.1  *(4.7+7.4)* |  | 4.8-6.6 | 6.0  *(2+4)* |  | 110-130 | 121  (45+76) |
| 100 | *FBM* | 40-55 | 12 | 152-167 |  | 3.5-4.5 | 4.1  *(1.2e+2.9)* |  | 11.6-13.2 | 12  *(3.8+8.2)* |  | 4.8-6.6 | 6.4  *(2+4.4)* |  | 110-130 | 124  (40+84) |
| 110 | *FBM* | 30-45 | 12 | 152-167 |  | 3.5-4.5 | 4.1  *(0.9e+3.2)* |  | 11.6-13.2 | 11.8  *(2.8+9)* |  | 4.8-6.6 | 6.8  *(2+4.8)* |  | 110-130 | 127  (35+92) |
| 120 | *FBM* | *Stop SPN unless clinically indicated* | | |  | 3.5-4.5 | 3.5  *(0+3.5)* |  | 11.6-13.2 | 9.8  *(0+9.8)* |  | 4.8-6.6 | 5.3  *(0+5.3)* |  | 110-130 | 101 |

*DOL* day of life, *PN* parenteral nutrition, *EN* enteral nutrition, *FBM* fortified breastmilk, *e* equivalent amino acid or protein (conversion 1g protein = 1.13g amino acid), *SPN* standardised parenteral nutrition.

^a^PremSmart-1 was indicated for the first 48 h after birth; PremSmart-2 was indicated from 48 h onwards.

^b^SMOFlipid^®^ with Vitamins (Fresenius Kabi, Graz, Austria), 1 g=6 mL.
