# Supplementary material for: Barriers and facilitators to the national scale‐up of a preterm standardised parenteral nutrition system: A mixed‐methods evaluation
Source: JPGN Rep. 2026 Jul 31:10.1002/jpr3.70213. Online ahead of print. doi: 10.1002/jpr3.70213 (PMC13425788; doi:10.1002/jpr3.70213)
Supplement: Supplementary file 9 — Suppl_Table_S3. [file JPR3-9999-0-s009.docx]

**Supplementary Table 3 Framework Method Analysis Coding Concepts**

**Table a. Stage 3 concepts**

| **A priori concept** | **CFIR Domain** | **CFIR Construct** | **Operational definition** |
| --- | --- | --- | --- |
| Perception of the system | Innovation | Innovation Relative: Advantage/Source /Evidence Base/Design | Reference to perceptions about the SPN system and reasons and sentiment on perception |
| Intervention fit | Innovation | Innovation Adaptability | Includes descriptions of necessary adaptations to improve fit. |
|  | Inner Setting | Compatibility | Reference to compatibility or not of the intervention with existing clinical norms, guidelines, practices, or perceived workflows |
| Audit and evaluation | Process | Reflecting & evaluating | Refers to ongoing measurement and feedback regarding implementation efforts. |
| Training and support | Inner setting | Access to knowledge and information | Guidance and/or training is accessible to implement and deliver innovation |
| Staff turnover | Inner Setting | Structural Characteristics: Work infrastructure | Use this code when participants describe the departure, replacement, or loss of staff and it actual or perceived impact on implementation/sustainment of the intervention. |
| Staffing levels | Inner Setting | Structural Characteristics: Work infrastructure | Directly relates to human resource capacity for implementation. |
| Team formation | Inner Setting | Structural Characteristics: Work infrastructure | Depending on context, could reflect structural aspects (team org) - who supports PN delivery on a day-to-day basis. |
| Team Relationship | Inner setting | Relational connections | Directly pertains to the quality and strength of informal/formal connections between team members |
| National Groups - Expert Group/Implementation Team | Outer setting | Partnerships and Connections | Reference to linkages with national groups |
| System as ‘National Model of Care’ | Outer setting | Policies and Law | Reference to national system – mandatory guideline. |
| Funding | Outer setting | Financing | Reference to funding for implementation and sustainment |
| Role of Champion | Individual Domain | Implementation Lead/Team Members | Reference to local or national implementation leaders or team members |
| Leadership | Individual Domain | Mid-level leader | Reference to leadership e.g. Consultants who may impact on the implementation or sustainment of the intervention |
| Miscellaneous | n/a | n/a | Any relevant item inductive coded for discussion with team – include memo with code |

**Table b. NVivo Codebook\\Stage 3 Systematic Coding**

| Name | Description | Sources | References |
| --- | --- | --- | --- |
| Audit and Evaluation | Refers to ongoing measurement and feedback regarding implementation efforts | 12 | 56 |
| Behaviour | Capability, Motivation or Opportunity to enact a specific behavior | 12 | 135 |
| Capability |  | 9 | 41 |
| Motivation | The individual is committed to fulfilling the role | 7 | 26 |
| Opportunity | availability, scope and power to fulfil role | 8 | 17 |
| Funding | National or local financing | 2 | 3 |
| Illustrative Quote | This code is for quotes that are particularly illustrative of codes or important aspects of the research. | 6 | 12 |
| Intervention fit | Compatibility with existing practices including descriptions of necessary adaptions to improve fit. | 20 | 208 |
| Compatibility | Reference to compatibility or not of the intervention with existing practice | 20 | 127 |
| Innovation Adaptability | Includes descriptions of necessary adaptations to improve fit. | 16 | 78 |
| Leadership | References to local or national leadership that help or hinder the implementation and sustainment of the intervention | 11 | 37 |
| Miscellaneous | Any relevant item - inductively coded and for further discussion with the team | 5 | 10 |
| National - Outer Setting |  | 0 | 0 |
| National Groups | Reference to linkages or impact of national groups - National PN expert group, National Implementation Team | 12 | 23 |
| National Model of Care | Impact of this being a national guideline - i.e. policy or law that it is required to follow | 5 | 13 |
| Perception of the system | References to perception about the SPN system and reasons for same | 18 | 195 |
| Design |  | 12 | 46 |
| Evidence Base |  | 7 | 17 |
| Relative Advantage |  | 8 | 13 |
| Source |  | 2 | 7 |
| Phase | Phase of implementation - initial implementation or sustainment phase | 0 | 0 |
| Initial Implementation |  | 16 | 73 |
| Sustainment |  | 16 | 155 |
| Role of the Champion | References to local or national implementation leads or team members | 17 | 63 |
| Training and Support | guidance and/or training is accessible to implement and deliver the innovation | 20 | 257 |
| Workforce | Staffing levels, turnover and teaming | 20 | 289 |
| Staff levels | Human resources capacity for implementation or sustainment of intervention | 12 | 38 |
| Staff turnover | Departure, replacement or loss of staff and its actual or perceived impact on implementation or sustainment of the intervention | 15 | 48 |
| Team formation | Aspects of team organisation - who is available on a day-to-day basis to support PN delivery. | 18 | 114 |
| Team Relationship | Pertains to the quality and strength of informal/formal connections between team members. | 16 | 87 |

**Table c. NVivo Codebook\\Stage 4 Developing a working analytical framework**

| Name | Description | Sources | References |
| --- | --- | --- | --- |
| Audit & Evaluation | Refers to ongoing measurement and feedback regarding implementation efforts | 12 | 56 |
| COM-B | Capability, Motivation or Opportunity to enact a specific behaviour | 17 | 156 |
| Capability |  | 10 | 43 |
| Motivation | The individual is committed to fulfilling the role | 15 | 45 |
| Opportunity | availability, scope and power to fulfil role | 8 | 17 |
| Ilustrative Quote | This code is for quotes that are particularly illustrative of codes or important aspects of the research. | 12 | 21 |
| Innovation - context fit | Compatibility with existing practices including descriptions of necessary adaptions to improve fit. | 20 | 211 |
| Compatibility | Reference to compatibility or not of the intervention with existing practice | 20 | 128 |
| Innovation Adaptability | Includes descriptions of necessary adaptations to improve fit. | 16 | 80 |
| Miscellaneous | Any relevant item - inductively coded and for further discussion with the team | 5 | 10 |
| Perception of the Innovation | References to perception about the SPN system and reasons for same | 18 | 200 |
| Design |  | 12 | 46 |
| Evidence Base |  | 8 | 18 |
| Relative Advantage |  | 10 | 15 |
| Source |  | 4 | 9 |
| Phase | Phase of implementation - initial implementation or sustainment phase | 0 | 0 |
| Initial Implementation |  | 16 | 73 |
| Sustainment |  | 16 | 155 |
| Policy, governance and professional networks | Reference to linkages or impact of national groups governance, advisory and professional groups - National Women and Infants Health Programme, National PN expert group, National Implementation Team | 12 | 35 |
| Role of the Champion | References to local or national implementation leads or team members | 17 | 64 |
| Training and Support | Guidance and/or training is accessible to implement and deliver the innovation | 20 | 259 |
| Formal Training | Formal training session given at implementation or during sustainment - i.e. classroom-based sessions given to rotating doctors in training. Includes online training module | 3 | 7 |
| On-the-job support from colleague | On the job support to learn about or use the innovation from a colleague | 4 | 4 |
| Other supports | Includes non-training or personnel support e.g. protocol availability, physical aids such as ready reckoners, digital version of the protocol | 1 | 2 |
| Workforce and Team functioning | Staffing levels, turnover and teaming | 20 | 316 |
| Relational aspects of the team | Pertains to the quality and strength of informal/formal connections between team members. | 16 | 97 |
| Team Coordination & Collaboration | Aspects of team organisation - who is available on a day-to-day basis to support PN delivery. | 18 | 126 |
| Workforce capacity | Human resources capacity for implementation or sustainment of intervention. | 12 | 43 |
| Workforce turnover | Departure, replacement or loss of staff and its actual or perceived impact on implementation or sustainment of the intervention | 15 | 48 |
