# Supplementary material for: Barriers and facilitators to the national scale‐up of a preterm standardised parenteral nutrition system: A mixed‐methods evaluation
Source: JPGN Rep. 2026 Jul 31:10.1002/jpr3.70213. Online ahead of print. doi: 10.1002/jpr3.70213 (PMC13425788; doi:10.1002/jpr3.70213)
Supplement: Supplementary file 10 — Suppl_Table_S4. [file JPR3-9999-0-s002.docx]

**Comparison of respondents’ characteristics by Sustainment and Usability**

|  | |  |  |  |  |  |  |
| --- | --- | --- | --- | --- | --- | --- | --- |
|  | Sustainment^1^ | | |  | Usability^2^ | | |
| Variable | n | median (IQR) | p-value^3^ |  | n | median (IQR) | p-value^3^ |
| Overall | 152 | 3.7 (3.3–4) |  |  | 137 | 75 (65–83.8) |  |
| Gender |  |  | 0.570 |  |  |  | 0.670 |
| Male | 26 | 4 (3.3–4) |  |  | 25 | 75 (68.8–85) |  |
| Female | 125 | 3.7 (3.3–4) |  |  | 111 | 75 (65–85) |  |
| Professional role |  |  | 0.977 |  |  |  | 0.024 |
| Nurse | 71 | 3.7 (3.3–4) |  |  | 63 | 72.5 (55–80) |  |
| Doctor in Training | 27 | 3.7 (3.3–4) |  |  | 20 | 81.3 (72.5–91.9) |  |
| Consultant | 30 | 3.7 (3.3–4) |  |  | 30 | 75 (66.9–75.6) |  |
| Dietician | 13 | 4 (3–4) |  |  | 13 | 82.5 (68.8–93.8) |  |
| Pharmacist | 11 | 4 (3.3–4) |  |  | 11 | 75 (67.5–80) |  |
| Unit level |  |  | 0.002 |  |  |  | 0.011 |
| Level 1 | 24 | 3.7 (3.4–4) |  |  | 21 | 72.5 (70–81.3) |  |
| Level 2 | 45 | 3.3 (3–4) |  |  | 42 | 71.3 (51.9–77.5) |  |
| Level 3 | 83 | 4 (3.3–4) |  |  | 74 | 75 (70–87.5) |  |
| Training received |  |  | <0.001 |  |  |  | 0.026 |
| Yes | 128 | 4 (3.3–4) |  |  | 118 | 75 (67.5–85) |  |
| No | 24 | 3 (2.8–3.7) |  |  | 19 | 65 (50–77.5) |  |
| Years of experience |  |  | 0.762 |  |  |  | 0.005 |
| 0-10 years | 69 | 3.7 (3.3–4) |  |  | 61 | 77.5 (72.5–87.5) |  |
| > 10 years | 83 | 3.7 (3.3–4) |  |  | 76 | 72.5 (60.6–76.9) |  |
| ^1^ possible range 0-4 with higher scores indicating greater sustainment | | | | | | | |
| ^2^ possible range 0-100 with higher scores indicating greater perceived usability | | | | | | | |
| ^3^from Mann-Whitney U test when two groups and from Kruskal-Wallis test when more than two groups | | | | | | |  |
